# Purinergic signaling: A gatekeeper of blood-brain barrier permeation

**DOI:** 10.3389/fphar.2023.1112758

**Published:** 2023-02-07

**Authors:** Yuemei Wang, Yuanbing Zhu, Junmeng Wang, Longcong Dong, Shuqing Liu, Sihui Li, Qiaofeng Wu

**Affiliations:** Acupuncture and Moxibustion College, Chengdu University of Traditional Chinese Medicine, Chengdu, Sichuan, China

**Keywords:** purinergic signaling, blood-brain barrier, endothelial cells, P2Y receptors, P2X receptors, P1 receptors, CD39, CD73

## Abstract

This review outlined evidence that purinergic signaling is involved in the modulation of blood-brain barrier (BBB) permeability. The functional and structural integrity of the BBB is critical for maintaining the homeostasis of the brain microenvironment. BBB integrity is maintained primarily by endothelial cells and basement membrane but also be regulated by pericytes, neurons, astrocytes, microglia and oligodendrocytes. In this review, we summarized the purinergic receptors and nucleotidases expressed on BBB cells and focused on the regulation of BBB permeability by purinergic signaling. The permeability of BBB is regulated by a series of purinergic receptors classified as P2Y_1_, P2Y_4_, P2Y_12_, P2X4, P2X7, A_1_, A_2A_, A_2B_, and A_3_, which serve as targets for endogenous ATP, ADP, or adenosine. P2Y_1_ and P2Y_4_ antagonists could attenuate BBB damage. In contrast, P2Y_12_-mediated chemotaxis of microglial cell processes is necessary for rapid closure of the BBB after BBB breakdown. Antagonists of P2X4 and P2X7 inhibit the activation of these receptors, reduce the release of interleukin-1 beta (IL-1β), and promote the function of BBB closure. In addition, the CD39/CD73 nucleotidase axis participates in extracellular adenosine metabolism and promotes BBB permeability through A_1_ and A_2A_ on BBB cells. Furthermore, A_2B_ and A_3_ receptor agonists protect BBB integrity. Thus, the regulation of the BBB by purinergic signaling is complex and affects the opening and closing of the BBB through different pathways. Appropriate selective agonists/antagonists of purinergic receptors and corresponding enzyme inhibitors could modulate the permeability of the BBB, effectively delivering therapeutic drugs/cells to the central nervous system (CNS) or limiting the entry of inflammatory immune cells into the brain and re-establishing CNS homeostasis.

## 1 Introduction

**FIGURE 1:**
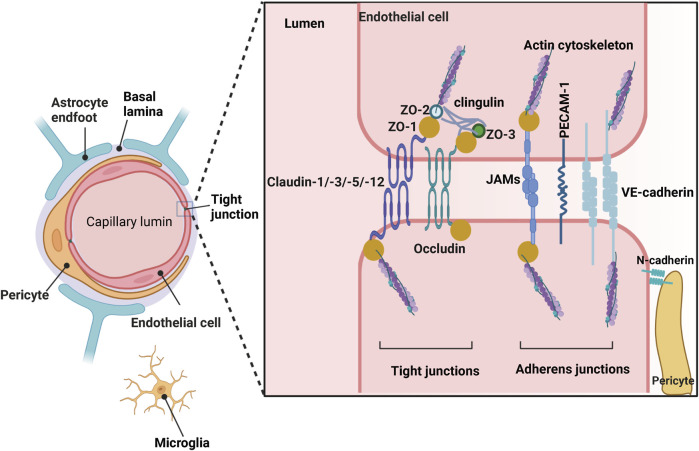
Structure of the blood-brain barrier (BBB) depicted graphically. Created with BioRender.com.

A well-developed central nervous system (CNS) barrier is very important for maintaining the homeostasis of the neural microenvironment. The blood-brain barrier (BBB) is a critical component of the CNS barrier and is composed of continuous endothelial cells within brain microvessels, which outline the physical structure of the BBB along with the end-feet of astrocytic glial cells, pericytes, and microglia (Kadry et al., 2020) (Figure 1). The BBB is the major site of blood-CNS exchange, controlling substances that can enter or leave the nervous tissue in a precise and tight manner. It also prevents harmful substances such as pathogens and toxins from entering the brain while allowing circulating nutrient substances from the blood to enter. BBB provides strong support for synaptic function, information processing, and neural communication, which explains why BBB is essential for maintaining the homeostasis of the intracerebral environment and peripheral blood.

BBB is highly selective for substances to cross; these distinct properties tightly control the delivery of ions, molecules, and cells between the blood and the neural microenvironment. BBB endothelial cells are connected by various molecular junctional complexes, including tight junctions and adherens junctions. Tight junctions (TJ) involve occludin and claudin-1, claudin-3, claudin-5, and claudin-12, ZO-1, ZO-2, and ZO-3, and adherens junctions involve cadherins, the platelet endothelial cell adhesion molecule (PECAM-1), and the junctional adhesion molecules (JAMs): JAMA, JAMB, and JAMC (Sweeney et al., 2019) (Figure 1). Oxygen, carbon dioxide, and small lipid-soluble molecules (weight <400 Da) or containing <8 hydrogen bonds (such as ethanol) could cross the BBB in transmembrane diffusion manner (Sweeney et al., 2019). Additionally, the BBB also provides a combination of specific ion channels and transporters on the abluminal membrane of the BBB to regulate the balance of ions in the brain, such as the sodium pump (Na^+^, K^+^-ATPase), regulating sodium influx into and exchange for potassium efflux out. In addition, ATP-binding cassette (ABC) transporters are concentrated on the luminal side of the BBB, mediating the movement of molecules such as drugs and xenobiotic agents (Dean et al., 2022). The existence of solute carrier-mediated transport (CMT) and receptor-mediated transcytosis (RMT) ensures the entry of macromolecules into the brain. CMT improves the transport of macromolecules like carbohydrates, amino acids, fatty acids, hormones, vitamins, et al., while RMT ensures the exchange of proteins and peptides between the blood and brain (Zhao et al., 2015; Sweeney et al., 2019).

Although the presence of the BBB protects the CNS from neurotoxic substances circulating in the blood, the BBB also prevents the transfer of most macromolecules (e.g., peptides, proteins, and nucleic acids), severely limiting the treatment of CNS diseases (e.g., neurodegenerative diseases, brain tumors, brain infections, and strokes). BBB breakdown has been identified as a critical component in several neurological conditions. It is reported that in clinical trials and animal experiments, BBB dysfunction promoted the progression of various CNS disorders such as Alzheimer’s disease (AD) (Cai et al., 2018; Zhao et al., 2021), multiple sclerosis (MS) (Niu et al., 2019), hypoxia and ischemia (Yang et al., 2018), and traumatic brain injury (TBI) (Dinet et al., 2019; van Vliet et al., 2020). Therefore, an in-depth dissection of the mechanisms to understand the basic properties of BBB is necessary to elucidate the development of the physiology and pathology of the CNS.

Purinergic signaling is essential in the CNS for maintaining the function of neurons, astrocytes, and microglia and controlling their homeostasis, consequently influencing synaptic transmission and higher cognitive processes (Burnstock, 2017; Burnstock, 2020; Illes et al., 2020). It has been demonstrated that several purinergic receptors are broadly dispersed throughout the CNS, being present in neurons, glial cells, and endothelial cells (Burnstock, 2007; Muhleder et al., 2020). The integrity of the endothelial barrier is affected by the action of extracellular adenosine triphosphate (ATP) and its metabolites adenosine diphosphate (ADP) and adenosine (ADO) on the purinoceptors of endothelial cells (Communi et al., 2000). Endothelial cells are capable of releasing nucleotides in response to a variety of physiological or pathological stimuli. Endothelial cells produce nucleotides in reaction to pathological stimuli including inflammation, hypoxia, blood flow fluctuations, shear stress, and changes in osmotic pressure (Gunduz et al., 2006; Hartel et al., 2007). Meanwhile, a growing number of studies have shown significant modulation of the endothelial barrier by purinergic substances or purinergic receptors, including modulation of BBB permeability (Wang et al., 2015; Chen et al., 2020; Wu et al., 2022). Here, we present current *in vivo* and *in vitro* investigations that implicate purinergic receptors and major metabolic enzymes as crucial regulatory routes for BBB permeability. Modulation of purinergic signaling, on the one hand, has effects on promoting its integrity and protecting the CNS from damage by peripheral harmful substances and, on the other hand, is expected to allow therapeutic drugs to cross the BBB effectively to reach the brain parenchyma and optimize the treatment of CNS diseases.

## 2 Purinergic system

Purinergic signaling was proposed by Geoffrey Burnstock in 1972, with the theory indicating that virtually all extracellular purines were involved in cell communication in all animals and humans (Burnstock, 1972). The four major purines, as important components of the purine system, are ATP, ADP, adenosine monophosphate (AMP), and adenosine. ATP supports intracellular energy storage and also serves as a neurotransmitter and signaling molecule for intercellular communication (Burnstock, 1972). The purinergic system also includes three key enzymes, named the ectonucleoside triphosphate diphosphohydrolases (E-NTPDases: NTPDase1/CD39), ectonucleotide pyrophosphatase/phosphodiesterases (E-NPP), and ecto-5′-nucleotidase (E-5′-nucleotidase/CD73). Extracellular ATP is broken down by metabolic enzymes to produce ADP, AMP, and adenosine, which may stimulate a series of purinergic receptors expressed on the cell surface (Abbracchio and Burnstock, 1994). Purinergic receptors are divided into two main categories, P1 and P2 receptors. The P1 receptor is composed of four adenosine-selective receptor subtypes: A_1_, A_2A_, A_2B_, and A_3_ receptors. P2 receptors mainly include P2X receptors and P2Y receptors. In detail, P2X receptors include P2X1-7, seven ligand-gated cation channel subtypes, and P2Y receptors contain P2Y_1, 2, 4, 6_ and _11–14_, eight metabolic G-protein-coupled receptor (GPCR) subtypes. Notably, P2X receptors respond only to ATP, whereas P2Y receptors respond to multiple nucleotides, including ATP/ADP, UTP/UDP, or UDP-glucose. Adenine-based nucleotides such as ATP are actively released by cells in the neurovascular unit, particularly astrocytes, microglia, and endothelial cells, which activate a variety of nearby purinergic receptors and induce changes in BBB barrier function (Burnstock and Knight, 2017; Lee et al., 2021).

The focus of purinergic research has been on adenine-based nucleotides and adenosine, while guanine-based components of this system have received comparatively less attention. Until now, there has been growing evidence of the extracellular effects of guanine-based purines. The nucleotides guanosine 5′-triphosphate (GTP), guanosine 5′-diphosphate (GDP), and guanosine 5′-monophosphate (GMP) constitute the guanine-based purines (GBPs). These purines are metabolized to guanosine by extracellular nucleotidases, and conversely, guanosine is converted to guanine by purine nucleoside phosphorylases (Rathbone et al., 2008).

Guanosine has been demonstrated to be neuroprotective in numerous *in vitro* and *in vivo* models of CNS illnesses, such as ischemic stroke, AD, Parkinson’s disease (PD), etc. (Su et al., 2009; Hansel et al., 2015; Lanznaster et al., 2017). The neuroprotective mechanisms of guanosine may involve the decrease of glutamatergic excitotoxicity to influence astrocyte function (Schmidt et al., 2007; Schmidt et al., 2008); modulation of the adenosinergic system (Almeida et al., 2017); as well as impacts on the inflammatory cascade response and oxidative stress (Paniz et al., 2014; Kovacs et al., 2015). However, there are few reports of guanosine-related purines directly regulating the integrity of the blood-brain barrier. In a rat model of cerebral ischemia, intranasal guanosine was delivered 3 h after stroke to prevent ischemia-induced motor impairment, brain cell death, and blood-brain barrier permeability (Muller et al., 2021).

Notably, it has been demonstrated that 3′-5′-cyclic guanosine monophosphate (cGMP), produced by GTP catalyzed by guanylate cyclase, disrupts the integrity of the BBB (Choi et al., 2018; Janigro et al., 1994; Chi et al., 1999). Although guanosine effects could open a new window in therapeutic approaches toward purinergic signaling in the CNS (Massari et al., 2021), due to the limited data on the effects of guanosine on the blood-brain barrier, this review focuses primarily on determining the association between adenine-based nucleotides, their receptors, and BBB permeability.

## 3 P2X receptors and signaling

P2X receptors belong to the family of ligand-gated ion channels, and seven subunits have been identified, namely P2XR (1–7). P2X receptors direct the inward flow of Ca^2+^, Na^+^, and K^+^ cations upon activation by extracellular nucleotides such as ATP (Shieh et al., 2006; Bernier et al., 2018). P2X receptors are widely distributed in tissues. P2X receptors in smooth muscle cells mediate fast excitatory junctional potentials, while in the central nervous system, activation of P2X receptors causes calcium ions to enter neurons and elicit neuromodulatory responses. Although the ATP-binding sites of P2X receptors are highly conserved, there are differences in ATP potency among the different isoforms (Illes et al., 2021). P2XR (1–6) receptors are active at low micromolar to submicromolar concentrations of ATP, whereas P2X7 receptors require hundreds of micromolar concentrations of ATP to activate.

### 3.1 P2X receptors in neurovascular unit (NVU)

Neuronal and perivascular microglia are in touch with endothelial cells, pericytes, and astrocytes, which form the neurovascular unit. All P2X receptor mRNAs and proteins have been detected in the endothelium of multiple vessels (Loesch and Burnstock, 2000; Glass et al., 2002; Ramirez and Kunze, 2002; Wilson et al., 2007), but the expression of these receptors is not completely uniform in the neurovascular units of the brain. Using immunocytochemistry and transmission electron microscopy, Andrzej Loesch found that P2X1 was predominantly expressed in the astrocyte end-foot of the rat cerebellar vascular neural unit, with no significant expression in cerebellar endothelial cells and pericytes (Loesch, 2021). Similarly, the perivascular component of glial cells in the cerebellum showed P2X4 receptor immunoreactivity, while it was unlabeled in endothelial and pericytes (Loesch, 2021). However, both microvascular endothelial and perivascular astrocytes in the hypothalamus were immunoreactive for P2X4 receptors, but positive expression of P2X4 receptors in pericytes was not observed (Loesch, 2021). In addition, P2X6 receptors were expressed mainly in rat paraventricular nucleus microvascular endothelial cells and perivascular astrocytes end-foot (Loesch, 2021). Human brain microvascular endothelial cells (HBMECs) express P2X7 receptors (Wang et al., 2022). After stimulation by LPS, intracellular mitochondria produced a large amount of ATP and activated P2X7R, which further mediated the activation of the intracellular Omi/HtrA2 apoptosis signaling pathway and promoted cell apoptosis (Wang et al., 2022). Although the expression of these receptors in neurovascular units is partially understood, how P2X receptors regulate BBB permeability is less well studied, and the regulation of BBB by receptors other than P2X4 and P2X7 is unclear. Table 1 shows the effects of P2X receptor agonists and antagonists on the BBB.

**TABLE 1:** Effect of P2 receptors agonist/antagonist on blood-brain barrier permeability.

Receptor/Agonist/Antagonists	Drug name	Model	Observation	Reference(s)
P2X4 antagonists	5-BDBD	Mouse ICH	Cerebral edema↓; Infiltrating leukocytes and microglia↓; Claudin-5↑; Extravasation of Evans Blue↓	Wu et al. (2022)
P2X4 antagonists	5-BDBD	Mouse	Neurological deficit scores↓; IL-1β↓; Infiltrating leukocytes↓; Microglia/monocyte activation↓; Extravasation of Evans Blue↓	Srivastava et al. (2020)
		MCAO		
P2X7 antagonist	A-804598	E-cigarette-induced BBB damage *in vitro*	Prevent BBB damage by improving mitochondrial dysfunction in endothelial cells	Mekala et al. (2022)
P2X7 antagonist	A-804598	LPS-induced BBB injury mouse	Hippocampal Occludin and ZO-1↑; Prevent BBB damage	Wang et al. (2022)
P2X7 agonist	BzATP	Human astrocytes and hCMEC/D3 coculture	ZO-1 and Occludin↓; IL-1β and MMP-9↑; 10 KDa dextran extravasation↑; Increased BBB permeability	Yang et al. (2016)
P2X7 antagonist	A-438079	Human astrocytes and hCMEC/D3 coculture	ZO-1 and Occludin↑; IL-1β and MMP-9↓; 10 KDa dextran extravasation↓; Prevent BBB damage	Yang et al. (2016)
P2X7 KO		Cecal ligation and puncture (CLP) mouse	Caspase-1 and MMP-9↓; breakdown product of ZO-1 and spectrin↓	Wang et al. (2015)
P2X7 antagonist	A-438079	Rat intracerebral hemorrhage (ICH)	Neurobehavioral deficits↓; brain water content↓; Evans blue extravasation↓; activated RhoA↓; Occludin, VE-Cadherin, ZO-1↑	Zhao et al. (2016)
P2X7 antagonists	Bright Blue G (BBG) and A-438079	MDMA-induced neuroinflammation in rats	MMP-9, MMP-3 activity↓, basal lamina degradation↓; IgG extravasation↓; microglial activation↓; Reduces BBB breakdown	Rubio-Araiz et al. (2014)
P2X7 antagonists	BBG	Rat model EAE	Clinical signs of EAE↓; claudin-5 and PDGFβR↑; Decreased BBB permeability	Grygorowicz et al. (2018)
P2Y_1_ antagonist	MRS2500	Hypoxic damage stimulated mouse primary brain microvascular endothelial cells *in vitro*	ZO-1 and VE-cadherin↑; Enhanced endothelial barrier integrity	Raghavan et al. (2022)
P2Y_4_ antagonist	Reactive Blue 2(RB-2)	Kainic acid-induced epileptic rat model	P2Y4/TSP-1/TGF-beta1/pSmad2/3 pathway↓, Evans Blue contents↓; Outflow of Alexa Fluor 488 from capillaries↓	Zhang et al. (2019)
P2Y_12_ antagonist/P2Y_12_ KO	P2Y_12_ antagonist clopidogrel	Laser-induced BBB opening in mice	Exhibited significantly diminished movement of juxtavascular microglial processes; outflow of Alexa Fluor 488 from capillaries↑; Aggravated BBB defects	Lou et al. (2016)

### 3.2 P2X receptors and blood-brain barrier

#### 3.2.1 P2X4

P2X4 is a typical P2X receptor, which can bind to P2X2, P2X5, and/or P2X6 to form heterotrimers (Antonio et al., 2014). It is expressed on the plasma membrane and in intracellular compartments. Meanwhile, P2X4 is a highly sensitive purinergic receptor that recognizes extracellular free ATP produced by dying cells following tissue injury, and is located in central and peripheral neurons, microglia, astrocytes, endothelial cells, and epithelial tissues (Montilla et al., 2020). The function of the P2X4 receptor in microglia has received extensive attention because of the relatively high level of expression of this receptor in these cells (Stokes et al., 2017). Microglia rely on migration and motility for active surveillance of the brain. P2X4 receptor activation drives microglia movement mainly through the phosphatidylinositol 3-kinase (PI3K)/Akt pathway (Ohsawa et al., 2007). Thus, microglia that move to the injured brain area express high levels of the P2X4 receptor (Domercq et al., 2013). P2X4 also contributes to the immune response of microglia, which affects BBB permeability in neuroinflammatory and degenerative diseases (Vazquez-Villoldo et al., 2014). After intracerebral hemorrhage (ICH), microglia activation and immune cell infiltration exacerbate cell death and BBB damage. P2X4R was shown to be overexpressed in the brains of ICH patients as well as in ICH animals. Its activation inhibited the secretion of anti-inflammatory cytokines from microglia after cerebral hemorrhage, which exacerbated inflammatory brain injury. Concomitantly, P2X4R inhibition with the selective inhibitor (5-(3-bromophenyl)-1,3-dihydro-2H-benzofuro[3,2-e]-1,4-diazepin-2-one (5-BDBD)) dramatically reduced cerebral edema, blood-brain barrier leakage in ICH animals by decreasing pro-inflammatory activity of microglia (Wu et al., 2022). In addition, 5-BDBD treatment significantly inhibited P2X4R expression in monocytes and microglia after ischemic stroke, while reducing neurological deficit scores, interleukin-1 beta (IL-1β) levels, and BBB permeability (Srivastava et al., 2020). Furthermore, P2X4R knockout reduced leukocyte infiltration into brain tissue and improved neurological function in an ischemic stroke model (Verma et al., 2017). Thus, P2X4 receptor regulation of microglia activity may be partially involved in the regulation of BBB permeability, but the in-depth mechanisms need further investigation.

#### 3.2.2 P2X7

ATP-induced proinflammatory effects of the P2X7 receptor have been extensively studied. The P2X7 receptor is best known for its effects on proliferation, apoptosis, and inflammation (Gu and Wiley, 2018). In addition, the P2X7 receptor (P2X7R) is of particular interest because of its association with BBB disruption (Andrejew et al., 2019). Recently, P2X7R has been implicated in alcohol and nicotine-induced BBB damage (Le Dare et al., 2019; Le Dare et al., 2021). Electronic-cigarette (E-Cig) vape (0% or 1.8% nicotine) decreased occludin and glucose transporter 1 (Glut1) protein expression in brain tissue and increased BBB permeability *in vivo* (Heldt et al., 2020). *In vitro* (Mekala et al., 2022), treatment of brain microvascular endothelial cells with ethanol (ETH), acetaldehyde (ALD), or 1.8% e-Cig elevated P2X7R and TRPV1 channel gene expression. Meanwhile, the P2X7R antagonist A804598 (10 µM) restored mitochondrial oxidative phosphorylation levels and played a protective role in preventing extracellular ATP release. BBB functional assays using trans-endothelial electrical resistance showed that blocking P2X7R channels enhanced barrier function. P2X7R antagonist may prevent alcohol or e-cigarette-induced BBB damage by improving mitochondrial dysfunction in endothelial cells. In addition, P2X7R plays a key role in LPS-induced BBB injury in mice. LPS significantly upregulated hippocampus P2X7R expression, whereas treatment with the P2X7R inhibitor A-438079 prevented the LPS-induced decrease in hippocampal Occludin and ZO-1 expression in mice (Wang et al., 2022).

**FIGURE 2:**
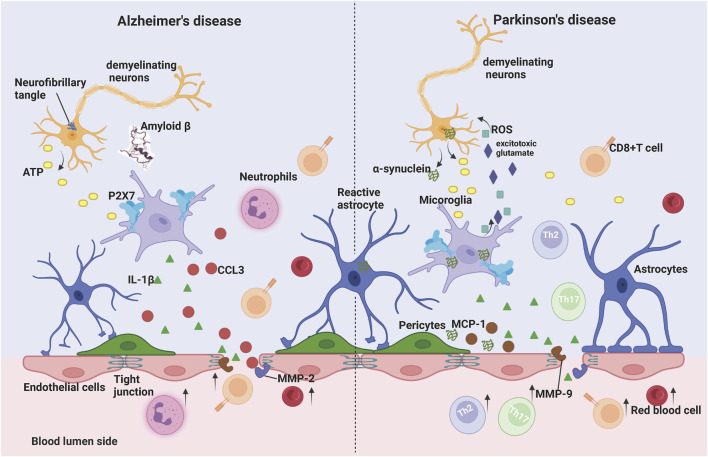
Microglia P2X7 receptor signaling on blood-brain barrier permeability in Alzheimer’s disease (AD) and Parkinson’s disease (PD). In AD, the process of neuronal death induces high levels of ATP release into the extracellular space, which activates microglia P2X7 receptors and leads to the release of IL-1β. In addition, the ATP/P2X7 receptor pathway enhances metalloproteinase (MMP) activity, leading to degradation of tight junction (TJ) protein, which results in increased blood-brain barrier permeability. Aβ peptide synergistically promotes P2X7 activation in microglia, which further induces increased release of chemokines such as CCL3 and recruitment of neutrophils and CD8^+^ T cells into the central nervous system (CNS) in the presence of BBB injury, promoting disease progression. In PD, α-synuclein aggregation leads to dopaminergic neuron death, increased ATP levels and P2X7 hyperactivation. On the other hand, α-synuclein activates microglia, leading to the release of excitatory glutamate and reactive oxygen species (ROS) from microglia to damage dopaminergic neurons, while α-synuclein binds to and stimulates the transcription of P2X7 receptors in microglia. α-synuclein and P2X7 receptors both increase the release of IL-1β and chemokines, increase MMP activity, disrupt the BBB, and lead to extravasation of red blood cells, which leads to cerebral microhemorrhage, as well as causing parenchymal infiltration of peripheral immune cells and exacerbating the pathological process of PD. Created with BioRender.com.

Microglia also contribute to the structural and functional integrity of the BBB. Although P2X7 receptors are expressed in a variety of cells in the brain, such as oligodendrocytes and astrocytes, their expression is highest in microglia (Illes et al., 2012; Illes, 2020). P2X7 is a receptor involved in microglial activation, which is significantly upregulated in the postmortem brain of Alzheimer’s patients and in animal models of various neurodegenerative diseases, promoting central neuroimmune and inflammatory responses that exacerbate disease progression (Takenouchi et al., 2010). Stimulation of P2X7Rs on the surface of microglia by high concentrations of ATP molecules activates NLRP3, which initiates the cleavage of pro-caspase-1 to caspase-1, followed by caspase-1-induced protein hydrolysis to convert pro-IL-1β to mature IL-1β. Studies have shown that P2X7 receptor activation of microglia leads to the release of the pro-inflammatory cytokine IL-1β (Ferrari et al., 2006; Takenouchi et al., 2009), which promotes the production of matrix metalloproteinase-9 (MMP-9) (Gu and Wiley, 2006) and decreases the expression of the tight junction protein ZO-1, thereby disrupting the blood-brain barrier *in vivo* and vitro (Harkness et al., 2000; Mori et al., 2002; Oliveira-Giacomelli et al., 2021). A breakdown in the blood-brain barrier resulted in neuroinflammation, ion dysregulation, and cerebral edema, leading to increased intracranial pressure, neuronal malfunction, and neurodegeneration (Profaci et al., 2020). For instance, extracellular aggregation of amyloid (Aβ) peptides is a key characteristic of AD, serving as an important trigger for glial cell activation and ATP release, thereby activating P2X7 receptors. Under AD pathology, high concentrations of ATP or Aβ peptides promoted the activation of P2X7 in microglia, which in turn induced increased release of chemokines such as CCL3 and the recruitment of CD8^+^ T cells into the hippocampus and choroid plexus and exacerbated the development of central inflammation (Martin et al., 2019) (Figure 2).

In addition, postmortem patients with PD have more phagocytically active reactive microglia in the brain (Toulorge et al., 2016), as well as increased microglia activation in the striatum and substantia nigra in a rat model (Carmo et al., 2014), accompanied by elevated P2X7 receptor gene expression (Oliveira-Giacomelli et al., 2021). α-synuclein protein is a crucial component of PD pathogenesis, and its aggregation is believed to be connected with disruption of the blood-brain barrier. Administration of α-synuclein dramatically increased the permeability of endothelium co-cultured with rat brain pericyte cells, while inducing the release of IL-1β, IL-6, TNF-α, MCP-1, and MMP-9 (Dohgu et al., 2019). Furthermore, α-synuclein protein activates microglia, causes the release of excitotoxic glutamate from microglia, and releases reactive oxygen species (ROS) to damage dopaminergic neurons (Dos-Santos-Pereira et al., 2018), as well as also binding to and stimulating transcription of P2X7 receptors in microglia (Jiang et al., 2015). Interestingly, P2X7 receptor activation increases IL-1β release, which in turn promotes MMP-9 secretion and disrupts the blood-brain barrier’s tight junctions (Yang et al., 2016). In PD disease, breakdown of the BBB causes extravasation of erythrocytes (Pienaar et al., 2015), which leads to cerebral microhemorrhages, as well as causing brain infiltration of peripheral immune cells and exacerbating the PD pathological process (Sweeney et al., 2018) (Figure 2).

**FIGURE 3:**
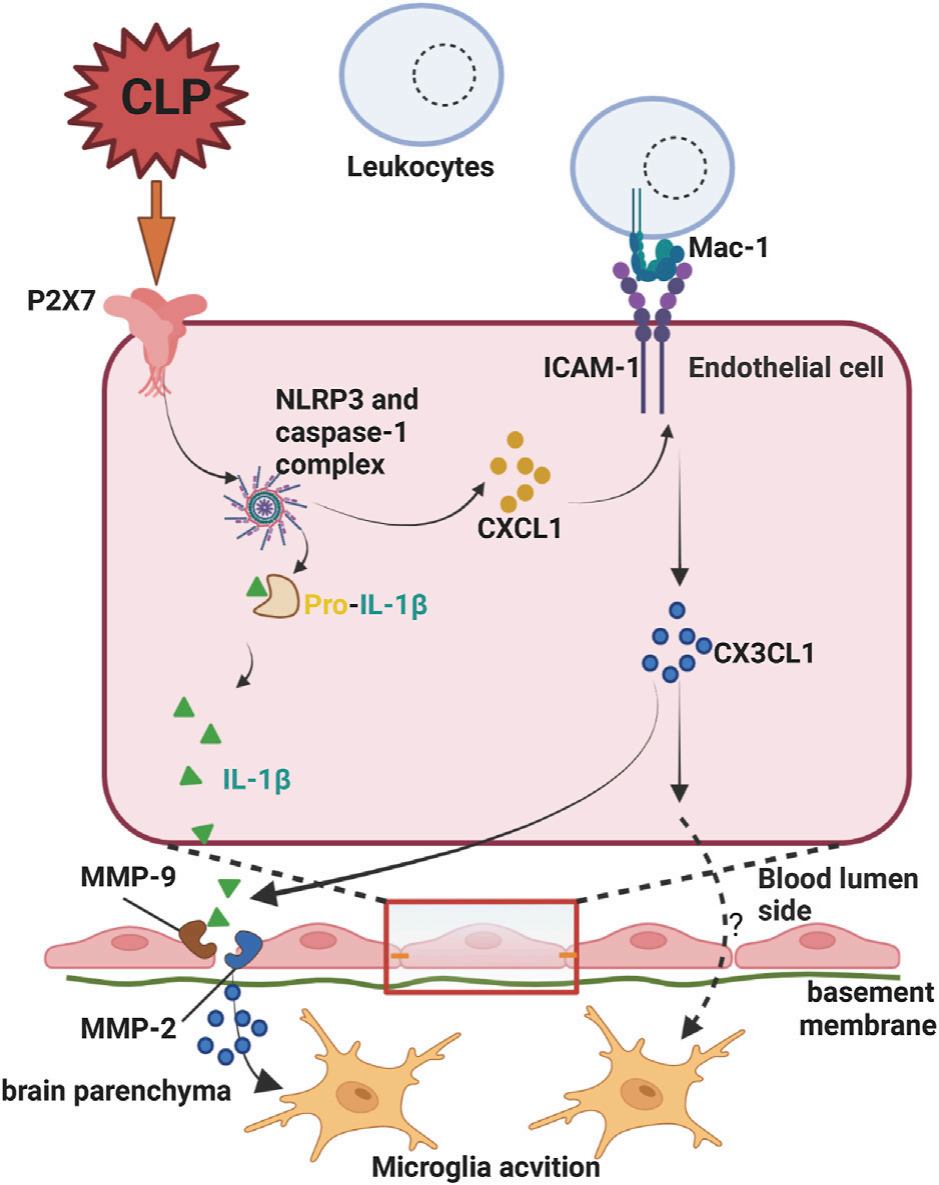
Endothelial P2X7 signaling on the permeability of the blood-brain barrier in septic encephalopathy (SE). Increased P2X7 receptor signaling in brain microvascular endothelial cells of SE mice generated by cecal ligation and puncture (CLP) increased adherence of Mac-1 expressing leukocytes to endothelial cells intercellular cell adhesion molecule-1(ICAM-1) and upregulated endothelial cell chemokine (C-X3-C motif) ligand 1 (CX3CL1), which activated microglia. The uncertainty exists as to whether CX3CL1 directly alters blood-brain barrier integrity during this process. P2X7 receptor signaling also promoted BBB breakdown and neurovascular injury during SE by activating NLRP3/caspase-1/IL-1β signaling. Created with BioRender.com.

P2X7 signaling in endothelial cells also plays a key role in BBB permeability regulation. Increased P2X7 receptor signaling in brain microvascular endothelial cells of septic encephalopathy (SE) mice induced by cecal ligation and puncture (CLP) enhanced the adhesion of Mac-1-expressing leukocytes to endothelial cells via intercellular cell adhesion molecule-1 (ICAM-1) and upregulated endothelial cell chemokine (C-X3-C motif) ligand 1 (CX3CL1), which triggered microglia activation. In addition, activation of NLRP3/caspase-1/IL-1β signaling *via* P2X7 receptor signaling in endothelial cells also accelerates BBB breakdown and neurovascular damage during SE (Wang et al., 2015) (Figure 3).

It has been determined that astrocytes participate in BBB function and can affect the permeability of the BBB (Bang et al., 2017; Heithoff et al., 2021). Formation and maintenance of the blood-brain barrier require astrocyte endfeet on the abluminal side of endothelial cells. In primary astrocytes, BzATP activates the P2X7 receptor and generates a 2.5-fold increase in RhoA activity, which is reduced by the P2X7 antagonist BBG, demonstrating that RhoA is activated in the signaling pathway downstream of the P2X7 receptor (Henriquez et al., 2011; Beckel et al., 2014). *In vivo*, increased expression of P2X7 receptors was observed in astrocytes and endothelial cells surrounding the hematoma 24 h after ICH in rats (Zhao et al., 2016). Disruption of the blood-brain barrier is one of the most significant pathophysiological alterations early in the course of ICH, leading to the production of vasogenic brain edema, which can result in a poor prognosis for the disease (Zhou et al., 2014). By suppressing RhoA activation, A438079, and P2X7R siRNA alleviated neurological impairments, brain edema, and minimized BBB degradation (Zhao et al., 2016). Numerous studies have identified RhoA, a small guanosine triphosphatase (GTPase), as a key regulator of barrier formation and disruption. RhoA governs endothelium actin cytoskeleton dynamics and contraction, affecting intercellular junctional complexes, vascular permeability, and signal transduction (Amado-Azevedo et al., 2014; Ramos and Antonetti, 2017). Activation of RhoA in endothelial cells promotes the onset of BBB barrier disruption, as evidenced by the fact that its activation leads to stress fiber formation associated with disruption of interendothelial junctions, thereby increasing paracellular flux (Beckers et al., 2010; Ramos and Antonetti, 2017).

The basement membrane (BM) is the extracellular matrix (ECM) that provides structural support for the BBB and also serves as a link between NVU intercellular communication and signaling pathways. It consists of structural proteins including type IV collagen, fibronectin, laminin, and other glycoproteins (Langen et al., 2019). P2X7 receptors are involved in the degradation of laminin and type IV collagen by the neurotoxic compound 3,4-methyldioxymethamphetamine (MDMA) (Rubio-Araiz et al., 2014). The intervention with MDMA activated microglia in the hippocampus and increased microglial P2X7 receptor expression. MDMA increased matrix metalloproteinase-3 (MMP-3) and MMP-9 activity in the hippocampus, which was accompanied by a decrease in laminin and collagen IV expression, an increase in IgG extravasation into the brain parenchyma, and finally lead to higher BBB permeability. Following treatment with P2X7 antagonists (Bright Blue G (BBG) and A-438079), BBB damage was minimized and its integrity was maintained (Rubio-Araiz et al., 2014; Perez-Hernandez et al., 2017).

Pericytes are in close contact with endothelial cells *via* “peg and socket” junctions in the common basal lamina, which are necessary for the formation and maintenance of the BBB (Cheslow and Alvarez, 2016; Liebner et al., 2018). One study has shown that P2X7R is co-expressed with PDGFβR, a pericyte marker localized to microvascular units (Grygorowicz et al., 2018). In a rat model of experimental autoimmune encephalomyelitis (EAE), P2X7 receptor expression was increased in capillaries, which correlated with low levels of expression of PDGFβR protein and Claudin-5. Treatment of P2X7R antagonists with immunized rats significantly reduced the clinical signs of EAE and enhanced the expression of claudin-5 and PDGFβR. These results suggest that P2X7 receptors located on pericytes may be involved in pathological mechanisms in brain microvessels that affect BBB integrity during EAE (Grygorowicz et al., 2018).

## 4 P2Y receptors and signaling

P2Y receptors are GPCRs with eight isoforms that respond to extracellular adenine and uracil nucleotides. P2Y receptors contain two subfamilies, the G_q_ protein-coupled P2Y_1_-like receptors P2Y_1, 2, 4, 6, 11_, and the G_i_ protein-coupled P2Y_12_-like receptors P2Y_12−14_. Almost all cells contain P2Y receptors, which are implicated in pathophysiological reactions like pain, inflammation, platelet aggregation, and neuroprotective effects (von Kugelgen, 2021). P2Y receptors are one of the most extensively researched therapeutic targets in the treatment of clinical diseases; e.g., clopidogrel, an antagonist targeting platelet P2Y_12_, is an anti-thrombogenic drug, and diclofosfamide, a nucleotide agonist targeting P2Y_2_ receptor, is used to treat dry eye disease (Guo et al., 2021).

### 4.1 P2Y receptors in NVU

P2Y receptors are mostly expressed in neurons, glial cells, and microvasculature in the brain, where they co-mediate neurotransmission, neuroprotection, neuron-glia interactions, and cerebral blood flow regulation alongside P2X receptors (Weisman et al., 2012; Toth et al., 2015; Burnstock, 2017). P2Y_1_ and P2Y_2_ receptors are present in brain pericapillary cells, and extracellular ATP causes pericyte contraction by stimulating these two receptors and causing intracellular Ca2^+^ concentrations to rise (Horlyck et al., 2021). P2Y_1_, P2Y_2_, and P2Y_13_ receptors, which are present in neurons, are involved in the regulation of neuronal differentiation and neuroprotection (Perez-Sen et al., 2015; Miras-Portugal et al., 2019). Notably, P2Y_1_, P2Y_2_, P2Y_4_, and P2Y_6_ receptors in endothelial cells have an induced vasodilation effect (Jacobson et al., 2020). Astrocytes in the hippocampus, cortex, striatum, cerebellum, and spinal cord express multiple P2Y receptors, such as P2Y_1, 4, 6, 13_ (Franke et al., 2012). Indeed, P2Y_12_ is relatively restricted in distribution, is mainly expressed in microglia, and has an important role in inflammation and neuropathic pain (Tozaki-Saitoh et al., 2008). The effects of P2Y receptors agonists or antagonists on BBB are shown in Table 1.

### 4.2 P2Y receptors and blood-brain barrier

Multiple P2Y receptor subtypes are expressed by endothelial cells throughout the vascular system. P2Y receptors in endothelial cells have been studied mainly in the context of their NO-mediated vasodilatory properties. Therefore, there are fewer findings on the role of P2Y receptors in maintaining the BBB. Bowden and Patel have identified the importance of the tyrosine kinase/mitogen-activated protein kinase (MAPK) cascades in P2Y receptor regulation of prostacyclin production in major vascular endothelial cells (Bowden et al., 1995; Patel et al., 1996). MAPK cascades are essential for cell adhesion, and there is substantial evidence that tyrosine-phosphorylated proteins are involved in maintaining the BBB integrity. Coexisting P2Y receptors in brain endothelial cells may variably control phosphoinositide hydrolysis, cyclic AMP, and MAPK, resulting in various effects on the BBB (Albert et al., 1997). The P2Y_1_ receptor exacerbates leukocyte recruitment and induces inflammation, and the P2Y_1_ inhibitor MRS2500 is able to reduce vascular inflammation. Meanwhile, in P2Y_1_-deficient mouse, monocyte adherence to inflammatory factor-stimulated mouse endothelial cell monolayers was drastically reduced *in vitro* (Zerr et al., 2011). Additionally, it has been discovered that primary cultured brain microvascular endothelial cells have minimal P2Y_1_ receptor expression and that hypoxic damage stimulated elevation of this receptor expression, which resulted in degradation of endothelial cell junctional proteins and increased endothelial permeability (Raghavan et al., 2022). Similarly, the P2Y_1_ receptor antagonist MRS2500 enhanced endothelial barrier integrity.

The development of epilepsy is accompanied by a disruption of BBB (Sweeney et al., 2019). The kainic acid-induced epileptic rat model presented angiogenesis and disruption of BBB integrity, along with a significant increase in the expression of TSP-1, TGF-β1, and pSmad2/3. Treatment with pyridoxal phosphate-6-azophenyl-2′, 4′-disulfonic acid (a broad P2 receptor antagonist) or Reactive Blue 2 (a P2Y_4_ receptor antagonist) inhibited TSP-1 expression and Smad2/3 phosphorylation level, while significantly reducing acute seizure severity, decreasing Evans Blue contents, and attenuating BBB damage (Zhang et al., 2019).

The P2Y_12_ receptor is a unique purinergic receptor expressed only by microglia in the central nervous system (CNS) (Gu et al., 2016). As a chemotactic receptor, it is highly expressed in microglia (Sasaki et al., 2003) and drives microglial migration to areas of CNS damage (Ransohoff and Cardona, 2010; Sipe et al., 2016). Following central capillary injury, perivascular microglia, a component of the neurovascular unit, rapidly generate dense aggregates of microglial protrusions at the site of injury. In addition, P2Y_12_ receptor-mediated chemotaxis of microglia processes is necessary for the rapid closure of the BBB after its rupture (Lou et al., 2016). Movement of paravalvular microglial protrusions was significantly reduced and failed to close the opening of the laser-induced BBB in mice intervened with the P2Y_12_ receptor inhibitor clopidogrel and in mice knocked out with the P2RY_12_ gene (Lou et al., 2016). Given that P2Y_12_ receptor antagonists are commonly used as platelet inhibitors in patients with coronary heart disease and cerebrovascular disease, who are at increased risk for stroke with impaired BBB destruction, these findings may have clinical implications.

## 5 Adenosine and adenosine (P1) receptors

Adenosine is a bioactive compound that has been shown to possess strong neuromodulatory effects. It is able to function as a signaling molecule between the body’s periphery and the brain since it can easily penetrate the BBB (Chiu and Freund, 2014). AMP is a major source of intracellular and extracellular adenosine. Intracellular adenosine is a synergistic intermediary between nucleic acids and ATP, which is generated by AMP metabolism via 5′-nucleotidase and synthesized by adenosine kinase. Adenosine can also be produced outside of the cells by the breakdown of ATP or ADP that has been released by the cell. In this pathway, CD39 or E-NTPDase converts ATP/ADP to AMP, while CD73 or 5′nucleotidase converts AMP to adenosine (Yegutkin, 2008). Adenosine exerts its effect by acting on four expressed G-protein-coupled adenosine receptors (A_1_, A_2A_, A_2B_, and A_3_) on cell surfaces, and these receptors are expressed in some combination on almost all CNS cells. Under physiological conditions, extracellular adenosine levels range between 20 and 300 nM (Newby, 1985; Newby et al., 1985); however, local adenosine concentrations in the brain increase nearly 1000-fold under stress and inflammatory conditions (Hagberg et al., 1987). A_1_ and A_2A_ receptors have a higher affinity for adenosine, in contrast to A_2B_ and A_3_ receptors, which have a lower affinity for adenosine, indicating that A_1_ and A_2A_ receptors in the CNS could be activated by reasonable levels of extracellular adenosine (Carman et al., 2011).

### 5.1 Adenosine receptors in NVU

The human brain endothelial cell line hCMEC/D3 exhibited A_1_, A_2A_, and A_2B_ receptors (Mills et al., 2011). A_1_ and A_2A_ receptors were also expressed in primary human brain endothelial cells and in Bend.3 mouse brain endothelial cells (Carman et al., 2011). In addition, *in vivo* immunofluorescence reveals that A_1_ and A_2A_ receptor proteins are expressed in mouse cortical brain endothelial cells, while *in vitro*, the two receptor proteins are present in primary mouse brain endothelial cells (Carman et al., 2011). Four P1 receptors (A_1_, A_2A_, A_2B_, and A_3_) have been identified in astrocytes (Dare et al., 2007). In astrocytes, cyclic adenosine monophosphate (cAMP) synthesis is inhibited by A_1_ receptors, while A_2_ receptors enhance cAMP synthesis, and A_2B_ receptors are able to lead to a dose-dependent accumulation of cAMP (Peakman and Hill, 1994). Adenosine receptors govern various features of astrocytes, including A_2A_ receptors that regulate glutamate uptake (Matos et al., 2012), but also A_1_ receptors that preserve cell integrity (Ciccarelli et al., 2001; D'Alimonte et al., 2007), and A_3_ receptors that protect against hypoxia-induced cell death and regulate CCL2 chemokine production (Wittendorp et al., 2004; Bjorklund et al., 2008). In physiological conditions, A_1_, A_2A_, A_2B_ and A_3_ receptors are moderately expressed in glial cells, but their levels are upregulated in a central inflammatory environment (Hasko et al., 2005). The blood-brain barrier could be altered with disruptive changes in endothelial cells and tight junctions during central chronic inflammation, mediated mainly by adenosine receptors and CD39/CD73 expression (Selmi et al., 2016). The effects of adenosine receptors agonists or antagonists on BBB are shown in Table 2.

**TABLE 2:** Effect of P1 receptors agonist/antagonist on blood-brain barrier permeability.

Receptor/Agonist/Antagonists	Drug name	Model	Observation	Reference(s)
A_1_ antagonist	DPCPX	*In vitro* BBB model:Co-culture of hCMEC/D3 and HEB	ERM and MLC phosphorylation↓; TEER↑; Na-F permeability↓; Improved BBB integrity	Liang et al. (2020)
A_1_ and A_2A_ agonists; A_1_ KO; A_2A_ KO	A_1_ agonists: CCPA; A_2A_ agonists: CGS 21680 or Lexiscan	Mouse model of brain drug delivery; mouse brain endothelial cells *in vitro*	*In vivo*:the permeability of the BBB to low molecule dextran↑; *In vitro*: TEER↓; Tight junction molecules↓; Actinomyosinstress fiber formation↑; A_1_/A_2A_ agonists induced BBB permeability, effects lost in KO mice	Carman et al. (2011)
Broad-spectrum adenosine receptors(AR) antagonist	Caffeine	MPTP-induced PD mouse model	Leakage of Evan’s blue dye and FITC-albumin ↓; ZO-1and occludin↑; Reactive gliosis↓	Chen et al. (2008a)
Broad-spectrum AR antagonist	Caffeine	Cholesterol-induced AD model in rabbits	Extravasation of IgG and fibrinogen↓; Leakage of Evan’s blue dye↓, Occludin and ZO-1↑; astrocytes activation and microglia density↓	Chen et al. (2008b)
A_2A_ antagonist	SCH58261	Sleep-restricted rats model	ZO-1 and claudin-5↑; 10 and 70 kDa FITC-dextran permeability↓; Evans blue permeability; GFAP and Iba-1 overexpression↓	Hurtado-Alvarado et al. (2016)
Adora2a ^ΔVEC^ mice		Thromboembolic stroke mice	Evans blue leakage↓; Leukocyte infiltration, brain edema, and neuroinflammation↓; NLRP3, caspase 1,IL-1β↓; Improved BBB integrity	Zhou et al. (2019)
A2A antagonist; Adora2a^ΔVEC^ mice	SCH58261	Diet-induced insulin-resistant mice	Evans blue and NaFl leakage↓; claudin-5 and Occludin↑	Yamamoto et al. (2019)
FDA-approved A_2A_ agonist	Lexiscan	Human brain endothelial barrier (hBBB) model *in vitro*	Phosphorylation of ERM and FAK↑; Claudin-5 and VE-Cadherin↓	Kim and Bynoe (2015)
A_2A_ agonist	CGS21680	Brain metastasis of lung cancer	Proliferation and migration ability of PC-9 cells↓; Claudin-5, Occludin, and ZO-1↑, MMP2 and MMP9↓ Improved BBB integrity	Chen et al. (2020)
A_2A_ agonist	CGS21680	*In vivo*: EAE model mice; *In vitro*: Th1 cytokine stimulation in mouse brain endothelial cells	MLC phosphorylation↓; ZO-1 and Claudin-5↑; Formation of stress fibers↓; Safeguard BBB function	Liu et al. (2018)
A_2B_ agonist	BAY 60–6583	Rats with (tMCAO)	Volume of tissue lesions↓; Brain swelling↓; leakage of albumin↓; MMP-9↓; ZO-1 degradation↓; Protects the blood-brain barrier	Li et al. (2017)
A_3_ agonist	AST-004	Mouse Model of Traumatic Brain Injury	Leakage of Evans Blue↓; ATP production in astrocytes↑	Bozdemir et al. (2021)

### 5.2 Adenosine receptors and blood-brain barrier

#### 5.2.1 A_1_ and A_2A_ receptors

**FIGURE 4:**
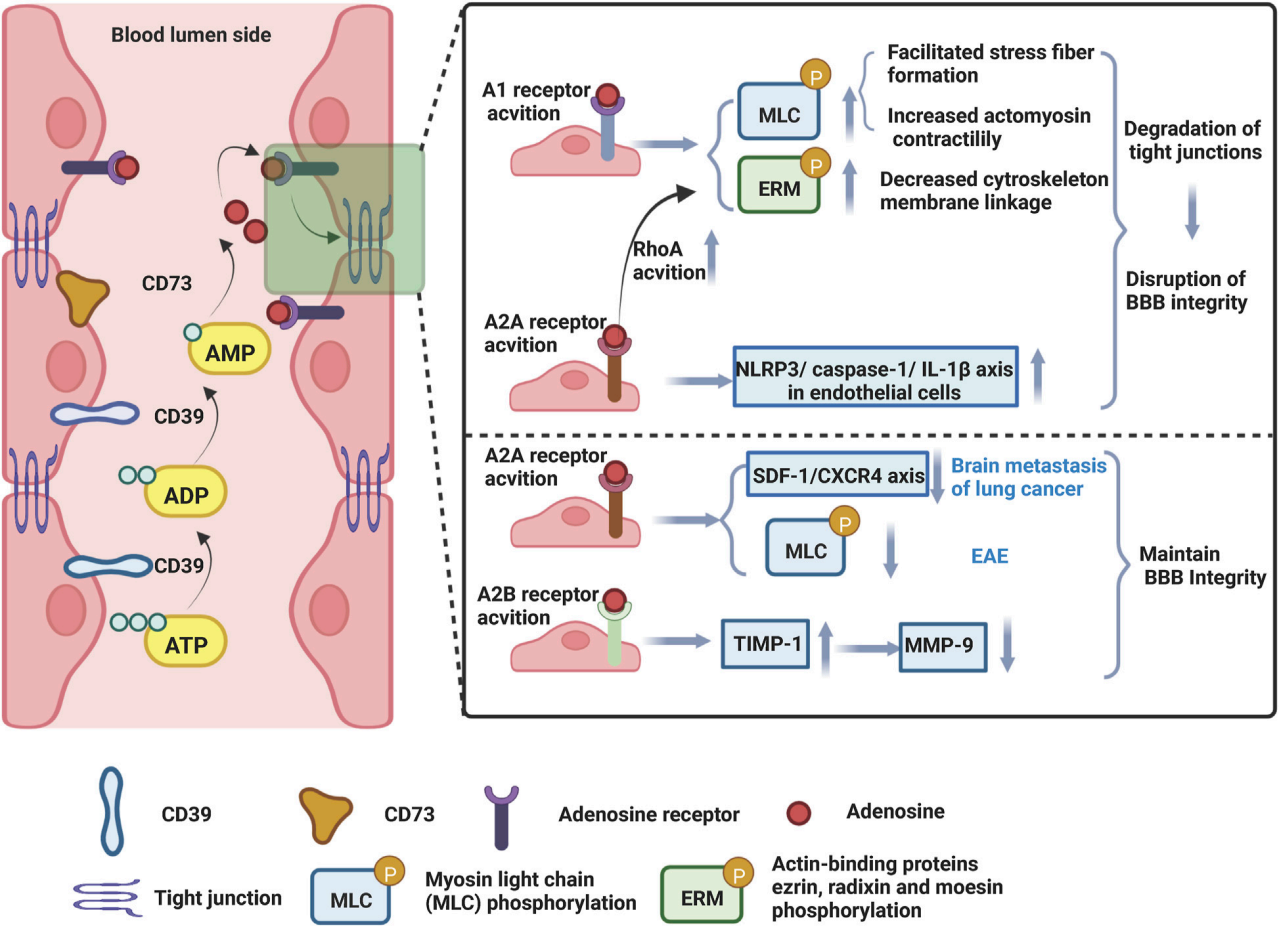
Adenosine receptors (ARs) modulate the permeability of blood-brain barrier. Endothelial cells of the microvasculature of the brain express adenosine receptors, CD39, and CD73. Under conditions of tissue damage, stress, and inflammation, ATP is released and converted by CD39 into ADP and AMP, which are then converted to adenosine by CD73. Adenosine binds to adenosine receptors on endothelial cells and operates to restructure the actin cytoskeleton, thereby disrupting tight junctions and adherens junctions and affecting BBB integrity. Created with BioRender.com.

Adenosine A_1_ and A_2A_ receptors are expressed on both human and murine brain microvascular endothelial cells (Kalaria and Harik, 1988; Carman et al., 2011; Mills et al., 2011). Two studies have shown that Ginkgo biloba extract increases BBB permeability by activating the A_1_ adenosine receptor signaling pathway (Guo et al., 2020; Liang et al., 2020). In these studies, BBB models were constructed by co-culture with human cerebral microvascular endothelial cells (hCMEC/D3) and human normal glial cells (HEB) *in vitro*. The hCMEC/D3 cell line is the first stable, well-differentiated human brain endothelial cell line that expresses CD73, a cell surface enzyme that converts extracellular AMP to adenosine, as well as adenosine receptor subtypes A_1_, A_2A_, and A_2B_ (Mills et al., 2011). Intervention with Ginkgo biloba extract increased BBB permeability, as evidenced by an increased fluorescein sodium (Na-F) penetration rate, disruption of tight junction structures, and increased actin-binding proteins ezrin, radixin and moesin (ERM) and myosin light chain (MLC) phosphorylation levels *in vitro* (Guo et al., 2020; Liang et al., 2020). ERM (ezrin/radixin/moesin) has been shown to be an important actin-binding molecule and the target of threonine phosphorylation in a variety of signaling pathways (Garcia-Ponce et al., 2015). Increased phosphorylation of ERM can induce actin remodeling and increase vascular permeability. Meanwhile, the phosphorylation of myosin light chain kinase (MLCK) to MLC causes actin filaments at the tight junction of endothelial cells to contract, leading to the opening of the barrier. Indeed, administration of the A_1_ receptor antagonist DPCPX or adenosine A_1_ receptor siRNA inhibited ERM and MLC phosphorylation levels, altered TJ ultrastructure, and improved BBB integrity (Guo et al., 2020; Liang et al., 2020) (Figure 4).

A_2A_ receptors are expressed at increased levels in brain tissue in multiple animal models, such as sleep restriction and thromboembolic stroke, accompanied by BBB damage (Hurtado-Alvarado et al., 2018; Zhou et al., 2019; Medina-Flores et al., 2020). Lack of sleep produces a low-grade inflammatory state that increases pro-inflammatory mediators, which regulate BBB function in a subtle but sustained manner (Hurtado-Alvarado et al., 2018). Sleep restriction increased the expression of A_2A_ adenosine receptors in the hippocampus and basal nucleus. Blockade of A_2A_ receptors by SCH58261 reversed sleep restriction-induced BBB dysfunction, including increased dextrans coupled to fluorescein (FITC-dextrans) and Evans blue permeability, degraded tight junction protein expression, and increased expression of neuroinflammatory markers Iba-1 and GFAP (Hurtado-Alvarado et al., 2016).

Besides, the human striatum contains high levels of A_2A_ receptors, while the cerebral cortex, hippocampus, and immune cells contain lower levels (van Waarde et al., 2018). It was demonstrated that the broad AR agonist NECA [activates all ARs (A_1_, A_2A_, A_2B_, A_3_)], the selective A_1_ agonist CCPA and the A_2A_ receptor agonists CGS 21680 or Lexiscan increased the permeability of the BBB to 10 kDa dextran, while the effect of these ARs on BBB permeability was attenuated in mice knocked out of A_1_ or A_2A_ receptors (Carman et al., 2011). Notably, *in vitro*, NECA and Lexiscan intervention in mouse brain endothelial cells inhibited the expression of intercellular tight junctions ZO-1, claudin-5, and Occludin and reduced transendothelial electrical resistance (TEER) (Carman et al., 2011). Consistent with these results, in animal models of neurodegenerative disease, investigators observed that caffeine, a broad-spectrum AR antagonist, attenuated Parkinson and Alzheimer animal models-induced leakage of Evans blue dye, degradation of occludin and ZO-1, and ameliorated BBB dysfunction (Chen et al., 2008a; Chen et al., 2008b). Likewise, the FDA-approved A_2A_ AR agonist Lexiscan, a selective A_2A_ receptor agonist, increased the permeability of the human brain endothelial barrier (hBBB) model *in vitro* (Kim and Bynoe, 2015). A_2A_ receptor activation mediated increase in cAMP and RhoA signaling activation, which in turn stimulated instability of the actin-cytoskeleton, decreased phosphorylation of factors involved in focal adhesion ERM and focal adhesion kinase (FAK) and degradation of Claudin-5 and VE-Cadherin, hence increasing the permeability of the hBBB (Figure 4). Notably, the high permeability of hBBB induced by A_2A_ agonists is rapid, time-dependent and reversible.

Accordingly, expression of A_2A_ receptors in endothelial cells was increased after thromboembolic stroke (Zhou et al., 2019). In mice specifically lacking endothelial Adora2a (Adora2a ^ΔVEC^), Evans blue leakage, leukocyte infiltration, brain edema, and neuroinflammation were attenuated. *In vitro* silencing of the Adora2a gene using siRNA in cultured brain microvascular endothelial cells also attenuated endothelial inflammation by inhibiting the NLRP3 inflammasome and downregulating expression of cleaved caspase 1 and IL-1β (Zhou et al., 2019). These results suggest that A_2A_ receptor-mediated NLRP3 activation may have a role in brain endothelial inflammation and need to be investigated in depth. It has also been shown that obesity and insulin resistance disrupt the BBB in both humans and animals (Banks, 2019; Banks and Rhea, 2021). The activation of A_2A_ receptors in endothelial cells is also closely associated with cognitive impairment caused by obesity. In this study, diet-induced insulin-resistant mice exhibited elevated BBB permeability to low molecular weight sodium fluorescein (NaFl) and Evans blue; however, administration of the A_2A_ antagonist SCH58261 restored the BBB barrier integrity (Yamamoto et al., 2019). SCH58261 is a selective adenosine A_2A_ receptor antagonist that crosses the BBB (Mohamed et al., 2012). Further study showed that Adora2a activation in endothelial cells exacerbated BBB damage and cognitive dysfunction in diet-induced insulin-resistant mice, while mice specifically knockout of endothelial Adora2a protected BBB integrity after suffering from diet-induced insulin resistance (Yamamoto et al., 2019). These results indicate that Adora2a-mediated signaling in vascular endothelial cells, which resulted in BBB failure, may be a potential mechanism for cognitive deficiencies caused by obesity and insulin resistance.

Certain viruses and bacteria infiltrated the CNS through boosting the local expression of adenosine, which promoted the BBB permeability and quickly opened the BBB (Zhao et al., 2020). For instance, *Haemophilus influenzae type a* (Hia) infection stimulated A_2A_ and A_2B_ adenosine receptors in a model of BBB co-cultured with human brain microvascular endothelial cells (BMEC) and pericytes (BMPC) *in vitro*, which induced the release of large amounts of VEGF from pericytes. VEGF caused pericyte shedding and endothelial cell proliferation, which triggered the BBB disorder (Caporarello et al., 2018). In addition, adenosine produced by a surface enzyme (Ssads) of *Streptococcus suis* promotes its pathogen’s entrance into the brains of mice, hence causing meningitis. A1 AR activation increases *S. suis* BBB penetration, and A_1_ AR signaling exploitation may represent a generic virulence mechanism (Zhao et al., 2020).

Conversely, some studies have shown that A_2A_ receptor activation also protects the integrity of BBB barrier. Brain metastases is the most common and lethal malignancy of the CNS, (Lowery and Yu, 2017; Singh et al., 2018), yet it is unknown how primary cancer cells traverse the BBB and metastasis to physiological regions of brain tissue. The A_2A_ receptor agonist CGS21680 inhibited the proliferation and migration ability of PC-9 cells, a type of lung cancer cell, and suppressed brain metastasis (Chen et al., 2020). Notably, activation of A_2A_ receptors *in vitro* increased the expression levels of Claudin-5, Occludin, and ZO-1, reduced the expression of MMP-2 and MMP-9, and increased the BBB integrity, whereas the opposite effect was obtained with the A_2A_ receptor antagonist SCH58261 (Chen et al., 2020). Stromal cell-derived factor-1 (SDF-1) is an important chemokine in homeostasis that interacts with C-X-C motif chemokine receptor 4 (CXCR4), which is commonly classified as a GPCR (Janssens et al., 2018). The interaction between SDF-1 and CXCR4 has been identified to regulate several cellular physiological processes, such as transcription, energy metabolism, cell adhesion, and chemotaxis (Mao et al., 2017). Their findings also showed that A_2A_ receptor stimulation inhibited CXCR4 expression and that A_2A_ receptor agonists and CXCR4 antagonists protected nude mice from the metastasis of malignant tumor cells *in vivo* and prolonged their survival time (Chen et al., 2020). Mechanistically, A_2A_ receptor activation maintained BBB integrity by regulating the SDF-1/CXCR4 axis, which in turn inhibited brain metastasis (Figure 4). Besides, specific A_2A_ receptor agonist CGS-21680 improved pathological and clinical manifestations of EAE by decreasing BBB permeability, inhibiting neuroinflammation (Liu et al., 2018). Th1 cytokines are known to activate MLCK, which promotes phosphorylation of MLC (p-MLC) and disrupts actin-myosin interactions, thereby regulating endothelial cell morphology (Capaldo and Nusrat, 2009). Thus, activation of MLCK also leads to TJ injury (Rissanen et al., 2013). *In vitro*, brain endothelial cells treated with the Th1 cytokines IL-1β, TNF-α, and IFN-γ exhibited barrier failure. By inhibiting MLC phosphorylation and promoting ZO-1 and Claudin-5 expression, the A_2A_ receptor-specific agonist CGS-21680 provided direct BBB protection (Liu et al., 2018). In addition, CGS-21680 helps maintain the shape of endothelial cells by reducing the formation of stress fibers in cells caused by Th1 cytokines (Liu et al., 2018). Activation of the A_2A_ receptor may safeguard BBB function by suppressing MLCK-mediated MLC phosphorylation in EAE (Figure 4). According to tissue injury and associated pathological conditions, activating A_2A_ receptors has both beneficial and detrimental effects in different diseases (Chen et al., 2013). It was shown that A_2A_ inhibited specific lymphocyte proliferation, reduced infiltration of CD4^+^ T lymphocytes, and suppressed inflammatory cytokine production, thus inhibiting EAE progression. Additionally, Lexiscan, an A_2A_ receptor-specific agonist, is an FDA-approved drug with proven therapeutic effects in inflammatory bowel disease, lung injury, and hepatic ischemia-reperfusion (Liu et al., 2016). Based on the complexity of the brain microenvironment, the activation of A_2A_ receptors may also have a dual effect on the function of the BBB.

#### 5.2.2 A_2B_ and A_3_ receptors

The interaction of A_2B_ receptors and A_3_ receptors with the BBB has been less reported. The A_2B_ receptor agonist BAY 60-6583 reduced the volume of tissue plasminogen activator (tPA)-induced lesions and attenuated brain swelling and BBB disruption in rats with ischemic stroke (Li et al., 2017). BAY 60-6583 inhibited tPA-enhanced MMP-9 activation, possibly by increasing the tissue inhibitor matrix metalloproteinase 1 (TIMP-1), thereby reducing TJ protein degradation and protecting the blood-brain barrier (Li et al., 2017) (Figure 4).

In a mouse model of traumatic brain injury, the adenosine A_3_ receptor agonist AST-004 decreased the permeability of BBB and neuroinflammation, and enhanced spatial memory (Bozdemir et al., 2021). Intervention with AST-004 boosted ATP production in astrocytes and enhanced neuroprotective efficacy after brain injury; however, the precise mechanism of enhancing BBB function requires additional investigation.

#### 5.2.3 CD39 and CD73

Ecto-5′-nucleotidase CD73 is an enzyme present on the cell surface that participates in the purine catabolism process and is capable of catalyzing the breakdown of AMP to adenosine. In the central nervous system, neurons, astrocytes, endothelium, and other cells release ATP, then CD39 catalyzes the conversion of ATP/ADP to AMP, and CD73 metabolizes AMP to adenosine (Burnstock and Boeynaems, 2014; Fuentes and Palomo, 2015). Multiple types of endothelial cells express CD39 and CD73 (Koszalka et al., 2004; Dudzinska et al., 2014), and expression of CD39/CD73 at the cellular level regulates tissue barrier function via modulating ATP levels (Colgan et al., 2006). CD73 is expressed in mouse (Bend.3) and hCMEC/D3 brain endothelial cell lines *in vitro* (Carman et al., 2011; Mills et al., 2011). Compared to human brain endothelial cells, mouse brain endothelial cell CD73 expression is extremely low *in vivo* (Mills et al., 2008). CD73 is highly expressed on choroid plexus epithelial cells that form the blood-cerebrospinal fluid barrier, but its expression is lower on brain endothelial barrier cells under steady-state conditions (Mills et al., 2008). However, CD73 was increased in the presence of cellular stress, local inflammation, or tissue injury that produced adenosine. Meanwhile, CD39 is widely expressed in brain endothelial cells (Wang and Guidotti, 1998), and CD39 on endothelial cells is conducive to reducing inflammatory cell transport and platelet reactivity, thereby reducing tissue damage after cerebral ischemia (Hyman et al., 2009). Extracellular adenosine is generated by ATP via metabolism of the CD39/CD73 extracellular nucleotidase axis and subsequently could regulate BBB permeability via adenosine receptor signaling expressed on BBB cells (Bynoe et al., 2015).

In BBB, CD73 expression is at a low level but is sensitive to cAMP through its promoter (Narravula et al., 2000). The released adenosine activates cell surface adenosine A_2B_ receptors, leading to reorganization of endothelial junctions and promoting barrier function (Narravula et al., 2000). In addition, interferon (IFN)-β treatment increased CD73 expression in human blood-brain barrier endothelial cells (BBB-EC) and human astrocytes, and upregulation of CD73 and increased adenosine production may contribute to the beneficial effects of IFN-β on multiple sclerosis(MS) by enhancing endothelial barrier function (Niemela et al., 2008). Grunewald et al. also demonstrated that *in vitro*, CD73 increased adenosine production and maintained cell shape and actin cytoskeleton stability, thereby reducing endothelial barrier permeability (Grunewald and Ridley, 2010).

## 6 BBB-permeable A_2A_-related compounds under clinical trials in neurodegenerative diseases

BBB-permeable purine-related compounds are primarily associated with A_2A_ receptors in clinical trials of neurodegenerative diseases. Caffeine is the most commonly consumed A_2A_ receptor antagonist that penetrates the blood-brain barrier. Caffeine enhances cognitive performance by decreasing hippocampus tau hyperphosphorylation, attenuating neuroinflammation, and reversing memory loss (Laurent et al., 2014; Carvalho et al., 2019; Jacobson et al., 2022), offering evidence for targeting A_2A_ receptors in the therapy of AD. The effect of caffeine on cognitive function in AD is being investigated in the Phase 3 clinical trial NCT04570085. Istradefylline is a potent selective A_2A_ antagonist that crosses the blood-brain barrier and has a high affinity for human A_2A_ receptors, improving dyskinesia in PD patients (Muller, 2015; Torti et al., 2018). Indeed, it has been used as a combination therapy with Levodopa (L-DOPA) in PD treatment. It was approved early in Japan and Korea and passed various clinical safety and efficacy tests in the United States in August 2019. In addition, Tozadenant (SYN115) was originally developed for the treatment of PD and has been studied as a monotherapy for PD and as a combination therapy with L-DOPA or dopamine agonists (Pourcher and Huot, 2015; Shang et al., 2021). In the initial clinical studies for the therapeutic efficacy of the decreased closure time, no major adverse effects were observed; however, seven cases of sepsis and six deaths occurred in 890 patients in phase 3 clinical trial (Lewitt et al., 2020). As a result, clinical development of this drug was terminated in 2018. Noteblely, Tozadenant analogues, including ^18^F-labeled radiotracers for prospective positron emission tomography (PET) imaging, have recently been identified as A_2A_ antagonists (Renk et al., 2021). Besides, vipadenant also belongs to a group of potent A_2A_ antagonists, which was used for the treatment of PD (Pourcher and Huot, 2015), but its development was stopped due to safety concerns and later switched to cancer immunotherapy (Yu et al., 2020). Table 3 summarized the BBB-permeable A_2A_-related compounds under clinical trials in neurodegenerative diseases, and some of these clinical trials have been completed while others are still ongoing.

**TABLE 3:** BBB-permeable A_2A_-related compounds under clinical trials in neurodegenerative diseases.

Compound	Company or sponsor (country)	Disease	Dose	Clinicaltrials.Gov number (phase)
Caffeine	University Hospital, Lille (France)	AD	200 mg	NCT04570085 (3)
			BID	
Istradefylline	Kyowa Hakko Bio (Japan)	PD	20 or 40 mg	NCT00250393 (2)
			BID	NCT00955526 (3)
				NCT01968031 (3)
Tozadenant	Hoffmann-La Roche (The Switzerland); Biotie Therapies (Finland)	PD	120 or 180 mg BID	NCT01283594(2)
				NCT03051607 (3)
Vipadenant	Vernalis (United Kingdom); Biogen Idec, RedoxTherapies (Juno Therapeutics) (US)	PD	30 or 100 mg	NCT00438607(2)
			QD	NCT00442780 (2)

## 7 Prospects for improving drug delivery within the CNS

The BBB strictly regulates the movement of ions, molecules and cells between the blood and brain cells and is essential for neurological function and protection. Despite the BBB’s protective function, it restricts the availability of therapeutic compounds to the brain, making it more difficult to treat illnesses of the central nervous system (Profaci et al., 2020). The emergence of drug modification modalities based on receptor-mediated transcytosis, neurotropic virus-mediated transport, nanoparticles and exosomes all provide solutions for crossing the BBB, and drugs supported by these technologies are currently being evaluated in multiple clinical trials (Zhou et al., 2018; Liu et al., 2021; Terstappen et al., 2021). Our literature review demonstrates that antagonists of P2X7 and A_2A_ receptors have beneficial therapeutic effects on brain damage, central inflammation, neurodegeneration etc., and attenuate the concurrent deterioration of the BBB barrier function. Up to now, a number of P2X7R antagonists that can cross the BBB have been developed (Bhattacharya, 2018; Wei et al., 2018). The compounds JNJ-47965567 and JNJ-42253432 demonstrated significant activity against P2X7R in rodents and humans, as well as effective BBB penetration. Likewise, novel blood-brain barrier permeable derivatives have been designed and synthesized as potential P2X7 antagonists known as compound 6 (2-(6-chloro-9h-purin-9-YL)-1-(2,4-dichlorophenyl) ethan 1-one), named ITH15004 (Calzaferri et al., 2021). It is a most potent, selective and highly BBB permeable antagonist and is considered to be the first non-nucleotide purine proposition for future drug optimization (Calzaferri et al., 2021). In addition, overexpression of A_2A_ receptor leads to progressive neurodegeneration, and A_2A_ antagonists have broad prospects for the treatment of CNS diseases. In the latest literature review, Merighi et al. used standard commercial software to calculate multi-parameter optimization (MPO) scores of CNS drugs for A_2A_ antagonists in clinical trials to predict the likelihood of the compound crossing the blood-brain barrier with appropriate metabolic stability (Merighi et al., 2022). Meanwhile, their analysis suggested that alkylxanthines caffeine and DMPX have good ability to cross the blood-brain barrier and are expected to be potential drugs for the treatment of CNS diseases (Merighi et al., 2022).

In addition, researchers concentrated on developing strategies to control the BBB in order to facilitate access to the CNS (Rajadhyaksha et al., 2011). Determining how to accomplish this in a safe and effective manner has a profound impact on the treatment of a variety of neurological conditions. Current interventions include the use of drugs such as mannitol or bradykinin analog (Cereport/RMP7) to induce disruption of barrier function. Hypertonic mannitol, reduces tight junction integrity through endothelial cell contraction (Dabrowski et al., 2021), but its limitation is that it may cause seizures (Marchi et al., 2007). Cereport/RMP-7 has shown some potential to transiently increase BBB permeability (Borlongan and Emerich, 2003) and has shown some efficacy in animal models for the treatment of CNS pathology, but has not yielded satisfactory results in clinical trials (Prados et al., 2003).

Agonists of some receptors are able to open the BBB to allow large molecules or cells to enter. The observation of transiently increased BBB permeability upon A_2A_ receptor activation suggests that exploiting this pharmacological effect holds the promise of facilitating drug delivery within the CNS (Carman et al., 2011; Kim and Bynoe, 2015). By labeling several copies of A_2A_ receptor-activating ligands on dendrimers, a sequence of nanoagonists (NAs) was produced. NAs tagged with varying amounts of AR-activating ligands can adjust the BBB opening time-window within a range of 0.5–2.0 h (Gao et al., 2014). The FDA-approved A_2A_ receptor agonist Lexiscan or the broad-spectrum agonist NECA increases BBB permeability and allows delivery of macromolecules to the CNS (Carman et al., 2011). Notably, there was a correlation between the duration of induced permeability and the half-life of the agonist. The half-life of BBB permeation induced by NECA intervention was 4 h. Its duration is substantially longer than that of Lexican, which induces BBB permeation with a half-life of 2.5 min (Carman et al., 2011). These findings suggest that the use of this agonist to briefly open the BBB may facilitate the delivery of therapeutic antibodies to the central nervous system. Since NECA is a broad-spectrum adenosine receptor agonist, additional research is necessary to better comprehend the mechanisms by which A_1_/A_2A_/A_2B_ receptors specifically mediate signaling involved in BBB permeability regulation and to optimize the parameters for drug design.

## 8 Conclusion

Extracellular nucleotides acting on purinergic receptors of BBB cells modulate the permeability of the blood-brain barrier, with different types of receptors and concentrations of nucleotides affecting the specific modulatory effects. Inhibition or knockdown of P2X4, P2X7, P2Y_1_, or P2Y_4_ receptors protect BBB barrier integrity, limiting the entry of toxic substances, inflammatory immune cells, etc. into the brain and conversely, inhibition of P2Y_12_ receptor further exacerbates BBB permeability. The P2X7 receptor is highly investigated in the regulation of BBB integrity, although the distribution of other P2 receptors in the NVU and the pharmacological effects of inhibitors of these receptors are comparatively less explored. Additionally, specific subtypes of P1 receptors also variably influence BBB permeability, with A_1_ and A_2A_ receptor activation boosting BBB permeability and A_2B_ and A_3_ receptor agonists protecting BBB integrity. Although a small number of studies have demonstrated a protective effect of A_2A_ agonists on BBB integrity, it cannot be overlooked that A_1_ and A_2A_ agonists transiently open the BBB to facilitate the passage of large or small molecules and that this process is reversible, with the BBB closing as the drug’s half-life passes, offering great promise for drug delivery to the CNS. Therefore, purinergic signaling, as the gatekeeper of the BBB, has a switching regulatory function on the BBB, and the intervention of appropriate agonist/antagonist of purinergic receptors is beneficial to restore CNS homeostasis under pathological conditions. Furthermore, purinergic receptors are widely distributed in NVU, and their regulation of the blood-brain barrier is intricate. However, studies on the regulatory effects of purinergic receptors on the BBB have primarily focused on endothelial cells, and the targeting of these receptors on other BBB cells such as pericytes is not well understood. For validation purposes, more *in vitro* BBB models and *in vivo* research targeting purinergic receptors on cells other than endothelial cells are required.
